# Whole genome sequencing identifies novel mutations in malaria parasites resistant to artesunate (ATN) and to ATN + mefloquine combination

**DOI:** 10.3389/fcimb.2024.1353057

**Published:** 2024-03-01

**Authors:** Gustavo Capatti Cassiano, Axel Martinelli, Melina Mottin, Bruno Junior Neves, Carolina Horta Andrade, Pedro Eduardo Ferreira, Pedro Cravo

**Affiliations:** ^1^ Global Health and Tropical Medicine (GHTM), Associate Laboratory in Translation and Innovation Towards Global Health, (LA-REAL), Instituto de Higiene e Medicina Tropical, (IHMT), Universidade NOVA de Lisboa, (UNL), Lisbon, Portugal; ^2^ BigOmics Analytics, Lugano, Switzerland; ^3^ Laboratory for Molecular Modeling and Drug Design (LabMol), Faculty of Pharmacy, Universidade Federal de Goiás, Goiânia, Brazil; ^4^ Laboratory or Cheminformatics (LabChem), Faculty of Pharmacy, Universidade Federal de Goiás, Goiânia, Brazil; ^5^ Center for the Research and Advancement in Fragments and Molecular Targets (CRAFT), School of Pharmaceutical Sciences at Ribeirão Preto, University of São Paulo, Ribeirão Preto, Brazil; ^6^ Life and Health Sciences Research Institute (ICVS), School of Medicine, University of Minho, Braga, Portugal

**Keywords:** plasmodium, drug resistance, artemisinin combination treatment, genomics, molecular dynamics simulations

## Abstract

**Introduction:**

The global evolution of resistance to Artemisinin-based Combination Therapies (ACTs) by malaria parasites, will severely undermine our ability to control this devastating disease.

**Methods:**

Here, we have used whole genome sequencing to characterize the genetic variation in the experimentally evolved Plasmodium chabaudi parasite clone AS-ATNMF1, which is resistant to artesunate + mefloquine.

**Results and discussion:**

Five novel single nucleotide polymorphisms (SNPs) were identified, one of which was a previously undescribed E738K mutation in a 26S proteasome subunit that was selected for under artesunate pressure (in AS-ATN) and retained in AS-ATNMF1. The wild type and mutated three-dimensional (3D) structure models and molecular dynamics simulations of the P. falciparum 26S proteasome subunit Rpn2 suggested that the E738K mutation could change the toroidal proteasome/cyclosome domain organization and change the recognition of ubiquitinated proteins. The mutation in the 26S proteasome subunit may therefore contribute to altering oxidation-dependent ubiquitination of the MDR-1 and/or K13 proteins and/or other targets, resulting in changes in protein turnover. In light of the alarming increase in resistance to artemisin derivatives and ACT partner drugs in natural parasite populations, our results shed new light on the biology of resistance and provide information on novel molecular markers of resistance that may be tested (and potentially validated) in the field.

## 1 Introduction

Human malaria parasites, especially *Plasmodium falciparum*, have evolved resistance to nearly all drugs available, including artemisinin (ART) derivatives. This class of compounds consists of rapidly acting drugs, with extremely high antimalarial potency (Kumar and Zheng, 1990; ter Kuile et al., 1993; Meshnick et al., 1996). The World Health Organization recommends their use in combination with longer lasting antimalarial partners, under the practice known as ACT (World Health Organization, 2015). Unfortunately, reports of parasites showing increased tolerance to one or both components of ACT are increasingly more frequent (Ward et al., 2022). If high levels of resistance to ACTs should evolve, the loss of the last effective treatment against the disease could become a reality.

ART derivative’s mode of action and the mechanisms of parasitic resistance in *P. falciparum* have been extensively studied. ART and its derivatives require the cleavage of their distinctive endoperoxide ring in order to be activated, after interacting with intraparasitic heme (Meshnick, 2002). Once activated, ARTs bind promiscuously to a large number of parasitic proteins (targets) that are important for several different metabolic pathways (Wang et al., 2015), causing parasite cell death through protein damage and disruption of essential cellular functions (Wang et al., 2017). Phenotypically, ART resistance in *P. falciparum* has been shown to correlate with a deceleration on the progression from the parasite’s ring stage, where heme occurs at low levels, to the trophozoite stage, where heme is abundant. The phenotype of ART resistance has been shown to be determined by several mutations in the propeller domain of the parasite kelch 13 (K13) gene, both through laboratory studies and in natural parasite populations (Ward et al., 2022). K13 mutants have been suggested to reduce the binding of ART to *P. falciparum* phosphatidylinositol-3-kinase (*Pf*PI3K), resulting in reduced *Pf*PI3K ubiquitination and a consequent lower synthesis of the phospholipid signaling molecule PI3P (Mbengue et al., 2015). Interestingly, PI3P has been implicated in intracellular trafficking events, including protein export and haemoglobin (Hb) endocytosis (Bhattacharjee et al., 2012), which is consistent with the observation that mutant ART-resistant parasites exhibit reduced endocytosis and haemoglobin uptake in ring stages (Behrens et al., 2021). More recently, mutations in the K13 gene were confirmed to confer artemisinin resistance by gene editing via clustered regularly interspaced short palindromic repeat (CRISPR)/CRISPR-associated protein 9 (Cas9) (Ghorbal et al., 2014; Simwela et al., 2020) or zinc finger nuclease (ZFN) (Straimer et al., 2015).

Our group has long been addressing both the genetics and genomics of ART and ACT resistance, using the rodent malaria parasite *Plasmodium chabaudi*. We first identified mutations in a gene coding for a deubiquitinating enzyme (UBP-1), which were shown to be selected for, in recombinant progeny from genetic crosses under ART treatment (Hunt et al., 2007), providing early evidence for a potential role of differential protein ubiquitination in ART resistance. Later, a parasite clone (*P. chabaudi* AS-ATNMF1), selected experimentally *in vivo* for resistance to artesunate (ATN) + mefloquine (MF) was shown to harbor amplification and overexpression of the *mdr1* gene (Rodrigues et al., 2010). Also, we have shown that *mdr1* duplications are consistently selected for in genetic crosses, when using not only artemisinin, but also other drugs such as mefloquine and lumefantrine (Borges et al., 2011). Later, we showed that an increased artemisinin-resistant phenotype occurred along with a mutation in a functional element of the AP2 adaptor protein complex (Henriques et al., 2013), suggesting that endocytosis and trafficking of membrane proteins may be involved.

In a bid to further explore ART resistance, and more specifically, the dynamics and genetics of resistance to ACTs, the genome of the ATN + MF-resistant AS-ATNMF1 clone was investigated genome-wide for the presence of mutations using second generation whole-genome sequencing. The mutations identified in AS-ATNMF-1 were also inspected in its ATN-resistant progenitor, AS-ATN. Among the variants found, one mutation in a 26S proteasome subunit is the most likely to modulate resistance to artemisinins in malaria parasites.

## 2 Materials and methods

### 2.1 Parasite clones, maintenance, and measurement of parasite growth

**Figure 1:**
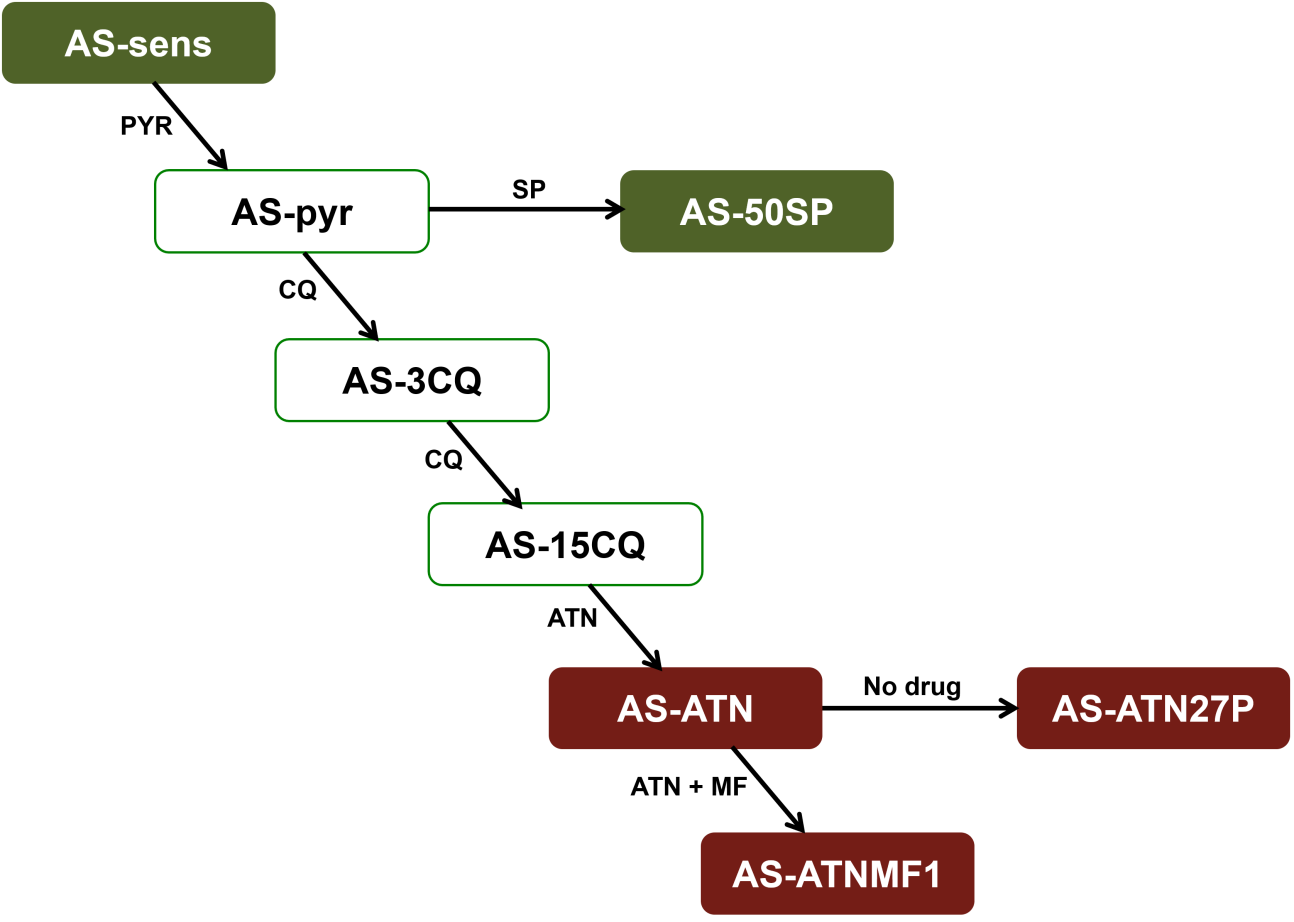
The AS lineage consists of an isogenic line of clones obtained a long time after many steps of selection with different antimalarials. These clones share a common precursor, the drug-sensitive clone, AS-sensitive. Drugs used for the selection of each clone are represented alongside each arrow: PYR, pyrimethamine; SP, sulphadoxine/pyrimethamine; CQ, chloroquine; MF, mefloquine; ART, artemisinin; ATN, artesunate. The clones used in this work are highlighted in red. In addition, genome sequences previously obtained for AS-sensitive and AS-50SP (highlighted in green) were used for filtering the lineage specific mutations identified by whole-genome sequencing of AS-ATNMF1.

Blood infected with *Plasmodium chabaudi* AS parasites was kept in liquid nitrogen and thawed on ice upon use. Infected blood was inoculated into six-to-eight week old CD1 male mice and parasite growth was observed during time by inspection of Giemsa-stained blood smears by optical microscopy. The parasite clones used in this study were (**Figure 1**): AS-ATN, which displays low level resistance to artesunate (Afonso et al., 2006); AS-ATNMF1, derived from AS-ATN through *in vivo* selection with ATN + MF administered simultaneously (Rodrigues et al., 2010); AS-ATN27P, a parasite that was obtained after twenty-seven consecutive sub-inoculations of AS-ATN into untreated animals (Rodrigues et al., 2010); clones AS-SENS (drug-sensitive) and AS-50SP (resistant to sulphadoxine and pyrimethamine).

All animal work was conducted according to relevant national and international guidelines: in Portugal, after approval by the Ethics Committee of the Instituto de Higiene e Medicina Tropical of Lisbon, Portugal, under PARECER 2/2006 from August 1^st^, 2006; in the United Kingdom, in compliance with the United Kingdom Animals (Scientific Procedures) Act 1986.

### 2.2 DNA extraction

Parasite DNA samples were extracted from blood by two different methods: (a) *Fresh blood samples* – Infected mice were exsanguinated by brachial artery puncture. Blood was processed, and DNA extracted as previously described (Grech et al., 2002). Host white blood cells were removed by CF11 cellulose (Whatman) and Plasmodipur filters (Eurodiagnostica); and (b) *Blood samples preserved in filter paper* – Blood samples were collected by a small puncture on the mouse tail vein. A drop was preserved in Whatman N° 4 filter paper and was extracted by boiling in Chelex-100 (Plowe et al., 1995).

### 2.3 Illumina^®^ (Solexa) genome sequencing

DNA obtained from fresh blood samples was processed using standard methods according to the manufacturer’s recommendation (Illumina, 2010). AS-ATNMF1 genome sequencing was performed using the Illumina^®^ (Solexa) platform, with 50 bp reads, using a paired-end read approach at the Genepool facilities at the University of Edinburgh, UK. Sequences were aligned against an isogenic reference genome [AS-WTSI, curated by the Pathogen Sequencing Group, Welcome Trust Sanger Institute and available at Sanger Institute webpage (Sanger Institute, 2009). The updated reference genome is available at https://plasmodb.org/plasmo/app/record/dataset/DS_5dab6e4a9f, with the following accession number: GCA_900002335.2] by two software packages, Mapping and Assembly with Quality (MAQ) (Li et al., 2008) and Sequence Search and Alignment by Hashing Algorithm (SSAHA2), using default settings (Ning, 2001).

SNP detection was performed using in-built algorithms, with a read depth of 3 set as the minimum threshold for SNP detection. In order to filter out SNPs arising in the sensitive progenitor AS-SENS, the list of SNPs obtained for AS-ATNMF1 was compared against a list previously obtained for AS-SENS (Hunt et al., 2010). The two filtered lists (for MAQ and SSAHA2) were then combined. Small indels (< 3bp) were detected using the internal algorithm of SSAHA2 only. The list of small indels was filtered against a similarly obtained list for AS-SENS (Hunt et al., 2010).

Larger indels (≥ 3bp) and copy number variation (CNV) detection was performed using SSAHA2 as previously described (Hunt et al., 2010). Two approaches were used to indicate areas of significantly decreased or increased coverage (Hunt et al., 2010). Since AS-ATNMF1 was sequenced using paired-end reads but AS-SENS was originally sequenced using single-end reads (Hunt et al., 2010), this approach could not be adopted using this clone, due to the excessive variation introduced by the different sequencing strategies adopted. Instead, a clone selected for sulphadoxine resistance (AS-50SP) and re-sequenced using paired-end reads (Martinelli et al., 2011) was selected to act as a “filter”, with the caveat that false positives and negatives are likely to appear. Due to a complete sequencing and analysis of other clones in the AS lineage (Hunt et al., 2010), we can rely on the data previously obtained for resolving any uncertainties and discovering false negatives. The positions of all the mutations described here are in accordance to an older version of the *P. chabaudi* annotated genome available on Welcome Trust Sanger Institute website as indicated above.

All SNPs were treated as potentially genuine mutations pending verification by di-deoxy sequencing. In the case of small and large indels, the vast majority is still expected to be false positive calls (Hunt et al., 2010; Martinelli et al., 2011; Kinga Modrzynska et al., 2012) and limited di-deoxy sequencing was performed based on the potential biological role attributed to the proteins coded by the genes where mutations were present or, in the case of intergenic mutations, the role of genes immediately close (either upstream or downstream) to the identified mutations.

### 2.4 PCR, di-deoxy sequencing and mutation inspection

A sub-set of mutations was chosen for verification by *di-deoxy* sequencing. For that purpose, a region of about a 1.000 bp flanking each mutation was used for designing oligonucleotide primers (**Supplementary Table 1**) and the fragments were amplified by PCR. DNA samples were obtained from dried blood spots and 1 μl of DNA was used as template in 50 μl reactions. The other reagents were added to 10x Green GoTaqR Flexi Buffer (Promega) to a final concentration of 1.5 mM MgCl_2_, 0.2 mM deoxynucleoside triphosphate (each), 0.2 pmol/μl forward and reverse primers, and 1.25 U of Go Taq Flexi DNA Polymerase (Promega). Cycling conditions were 93°C for 3 minutes, followed by 35 repeats of denaturation at 93°C for 30 seconds, annealing for 45 seconds, and elongation at 72°C for 1 minute. A final elongation step was performed at 72°C for 10 minutes. PCR products thus obtained were sequenced using the commercial services of STABVIDA Laboratories (Oeiras, Portugal). Chromatograms were inspected using Chromas 2.33 software (Technelysium).

### 2.5 Bioinformatics analysis of protein sequences

Predicted protein sequence and function was retrieved from PlasmoDB (Aurrecoechea et al., 2009) for all genes where a mutation was confirmed. Additionally, in cases where the predicted protein sequence did not contain a functional annotation in PlasmoDB, it was inspected for conserved domains and functional sites using InterProScan, a tool that combines different protein signature recognition methods into one resource (Zdobnov and Apweiler, 2001; Quevillon et al., 2005; Mulder and Apweiler, 2007).

### 2.6 Molecular modeling studies

The wild-type 3D structure of 26S proteasome subunit Rpn2 of *P. falciparum* (PF3D7_1466300) was built by threading approach through the I-Tasser server (Zhang, 2008; Yang et al., 2015). After modeling, the structure underwent refinement through the KobaMin server (Rodrigues et al., 2012), utilizing a knowledge-based potential of mean force for minimization (Summa and Levitt, 2007). Hydrogen atoms were added using the MolProbity server (Hintze et al., 2016). The mutant 3D structure E694K was derived from the wild-type structure by substituting the side chain of residue 694, using GROMACS v.5.1.2 (Van Der Spoel et al., 2005; Abraham et al., 2015). For the molecular dynamics (MD) simulations, the systems were solvated in a cubic box (solute box distance of 1.2 nm) and charge neutrality was achieved by introducing Na^+^ and Cl^−^ ions at a concentration of 0.15 mol/L. All simulations were conducted in GROMACS v.5.1.2, utilizing the AMBER FF99SB-ILDN force field (Cornell et al., 1995). Water molecules were described by the TIP3P model (Jorgensen et al., 1983).

The systems were prepared as mentioned above and then submitted to 1.000 steps of energy minimization using the steepest descent method, with harmonic restraints applied to all heavy atoms, to remove highly repulsive contacts or geometric strain. The minimizations were completed when the tolerance of 1000 kJ/mol was no longer exceeded. Subsequently, the following steps were performed to equilibrate the systems: (a) MD simulations using the NVT ensemble with harmonic restraints applied to all of the heavy atoms (1 ns); (b) MD simulations using the NPT (isothermal-isobaric) ensemble with harmonic restraints on all of the heavy atoms (1 ns); (c) MD simulations using the NPT ensemble without any restraints (1 ns). After these preparatory steps, MD trajectories of 100 ns for each system were generated as the production phase, in duplicates. The simulations were performed through periodic boundary conditions, using a cut-off of 1.0 nm, at a temperature of 300 K and 1 atm pressure. Electrostatics interactions were evaluated using the particle mesh Ewald algorithm (Leach, 2001). All RMSF and β-factor were calculated using the GROMACS gmx-toolbox (https://manual.gromacs.org/current/user-guide/cmdline.html). Visual Molecular Dynamics program (VMD) (Humphrey et al., 1996) was employed for visualizing MD trajectories, calculating residue-residue distances and rendering the molecular images. Additional molecular images were created using Python Molecular Viewer v.1.5.6 (Morris et al., 2009), while the graphs were generated using XMgrace software (http://plasma-gate.weizmann.ac.il/Grace/).

## 3 Results

### 3.1 Whole genome sequencing of AS-ATNMF1

The genome of the resistant clone AS-ATNMF1 was sequenced and 23,732,208 reads were produced with 21,816,439 reads (approximately 91% of all reads) mapped to the reference genome by SSAHA2. The average coverage of the whole genome was ~50-fold and 43% of the genome had at least 40-fold coverage, 92% was covered by at least 10 reads, whereas less than 0.7% of genome was not covered by any read. A subset of mutations was chosen for verification by di-deoxy sequencing, as will be described in detail below. It is worth noting that all the mutations analyzed by di-deoxy sequencing in AS-ATNMF1 and AS-ATN were also investigated in the parasites sub-inoculated twenty-seven times in untreated animals, AS-ATN27P. Unsurprisingly, AS-ATN27P showed the same genotype as AS-ATN.

#### 3.1.1 SNP detection

A total of 21 potential SNPs were identified by SSAHA2, which were all treated as potentially genuine mutations pending verification by di-deoxy sequencing. However, four SNPs were not subjected to di-deoxy sequencing. This was due to their location in subtelomeric regions or in unassigned contigs (bin), and the technical difficulties associated with PCR amplification of these regions. The remaining potential SNPs were successfully inspected by di-deoxy sequencing. Eight of these were rejected upon re-inspection as Illumina sequencing false positives (**Supplementary Table 2**).

The presence of nine genuine SNPs was confirmed by di-deoxy sequencing. Four of these had been previously identified: (a) V2697F substitution on the *pcubp1* gene (PCHAS_020720), coding for a deubiquitinating enzyme which has been associated with resistance to artesunate in the progenitor of AS-ATNMF1, AS-ATN (Hunt et al., 2007); (b) S109N replacement on the *pcdhfr* gene (PCHAS_072830), which encodes a dihydrofolate reductase enzyme previously implicated in pyrimethamine resistance (Hayton et al., 2002; Martinelli et al., 2011); (c) A173E substitution on the *pcaat1* gene (PCHAS_112780), coding for a putative amino acid transporter which appears to be involved in CQ resistance (Kinga Modrzynska et al., 2012); and (d) a T to G substitution in position 936,945 on chr 14, placed in an intergenic region, close to the 5´-end of the PCHAS_142600 gene of unknown function (annotated as *conserved Plasmodium protein*). This mutation was first identified in a clone showing resistance to PYR (Hunt et al., 2010; Martinelli et al., 2011).

Out of the five remaining genuine SNPs (**Table 1**), three were already present in the progenitor AS-ATN. These were (a) an A to G substitution identified in position 636,862 on chr 6. This nucleotide substitution falls within an intergenic region near the 3´-end of the PCHAS_061710 gene, which codes for a seryl t-RNA ligase, as indicated in PlasmoDB; (b) a K998* substitution on gene PCHAS_132020 whose corresponding product´s function is annotated as a conserved *Plasmodium* protein in PlasmoDB, but which contains an RNI-like signature following inspection through InterProScan; and (c) a E738K substitution on gene PCHAS_133430, coding for the 26S proteasome regulatory subunit RPN2.

**Table 1:** List of mutations identified exclusively in *Plasmodium chabaudi* AS-ATNMF1 and in its progenitor AS-ATN.

SNPs	Selected in	Type	Predicted protein function	Chromosome	Gene ID
a636,862g	AS-ATN	Intergenic	–	6	–
K998*	AS-ATN	Nonsense	Conserved *Plasmodium* protein in PlasmoDB. RNI-like Superfamily (aa 788-1421, 1.1E-13) in InterPro	13	PCHAS_132020
E738K	AS-ATN	Missense	26S proteasome regulatory subunit RPN2, putative	13	PCHAS_133430
K998L	AS-ATNMF1	Missense	Conserved *Plasmodium* protein in PlasmoDB. RNI-like Superfamily (aa 788-1421, 1.1E-13) in InterPro	13	PCHAS_132020
D560Y	AS-ATNMF1	Missense	Conserved *Plasmodium* protein in PlasmoDB. Armadillo-type fold in InterPro (aa 799-958, 1.5E-25)	14	PCHAS_143160

Only two SNPs were identified in AS-ATNMF1 as appearing after selection with the ATN + MF combination (**Table 1**). The first was a K998L mutation in the PCHAS_132020 gene. Interestingly, this gene was also mutated in the same residue in AS-ATN (which is the progenitor of AS-ATNMF1; see above), where a nucleotide substitution created a STOP codon (K998*), possibly generating a truncated form of the encoded protein. Thus, in AS-ATNMF1, the K998 mutation is likely to ‘rescue’ protein function either partially or completely (as the STOP codon present in AS-ATN was replaced by a leucine (neutral). In AS-SENS, this position originally coded for a lysine (positively charged).

Furthermore, we identified a D560Y mutation in PCHAS_143160. Although the function of the gene is unknown, inspection of its predicted protein sequence in InterProScan indicated that the product of this gene contains a region with high similarity to armadillo-type repeats (**Table 1**). These typically consist of a multi-helical fold comprised of two curved layers of alpha helices arranged in a regular right-handed superhelix, where the repeats that make up this structure are arranged about a common axis (Groves and Barford, 1999). Usually, these structures are well suited to binding proteins and nucleic acids and are found in a wide range of proteins.

#### 3.1.2 Indel and CNV detection

The 193 potential indels or CNVs were identified by SSAHA2 in AS-ATNMF1. As referred in the Material and Methods section, in this work only a sub-set of thirteen indels/CNVs was analyzed and, when possible, verified by di-deoxy sequencing (**Supplementary Table 3**). Ten of those were certified as false positives upon resequencing (**Supplementary Table 3**, highlighted in red). One indel identified on chr 3 in AS-ATNMF1 was confirmed by *di-deoxy* sequencing (**Supplementary Table 3**, highlighted in green) to be present also in its progenitor AS-ATN. This consisted of an AAT deletion (I103) which was identified in the PCHAS_031370 gene, a protein coding gene of unknown function, but predicted to have 12 transmembrane domains, according to PlasmoDB. Interestingly, the I103 deletion falls within the third predicted transmembrane domain of the protein and has been previously shown to be selected by chloroquine earlier in the AS lineage (Kinga Modrzynska et al., 2012).

A 34 bp deletion from position 876,894 to 876,927 on chr 7 present in AS-ATNMF1 (**Supplementary Table 3**, highlighted in blue) was not identified by Illumina sequencing due to the use of AS-50SP as a filter for the identification of indels but was verified by di-deoxy sequencing. This mutation first appeared in AS-PYR (Walliker et al., 1975), the progenitor of AS-50SP (Hayton et al., 2002). Instead, two SNPs very close to each other were identified (A to G substitution in position 876,917 and a C to A substitution in position 876,919 on chr 7). It is worth noting that SNP identification was made using AS-SENS as filter, instead of AS-50SP, and for that reason, these polymorphisms were detected and rather than being genuine SNPs were artefacts due to the presence of the deletion. The presence of this false negative raises the issue of other potential indels having been overlooked due to the use of AS-50SP as a filter. However, the extensive knowledge of the other clones belonging to the AS lineage minimizes the probability of other false negatives.

The remaining indels could not be verified by di-deoxy sequencing due to their large sizes or location in low complexity regions that prevent the design of suitable sequencing primers. However, there is strong evidence indicating they represented genuine polymorphisms, since large indels leave a strong signature in the reads mapping patterns.

These included deletions located on chr 5, chr 13 and contig 11844. The deletion on chr 5 was identified as present in other clones of the AS lineage in previous work (Hunt et al., 2010). A 60Kb deletion at the beginning of chr 13 was identified in AS-ATN (**Supplementary Table 3**), encompassing approximately 15 genes. A large deletion on contig 11844, which contains many features associated with sub-telomeric and telomeric chromosomal regions, was postulated to be a continuation of the sub-telomeric 60 kb deletion on chr 13. Indeed, contig 11844 has been merged with the sub-telomeric end of chr13 containing the deletion in the latest version of the *P. chabaudi* genome (Aurrecoechea et al., 2009; Brugat et al., 2017). The potential contribution of these deletions to artesunate resistance remains to be investigated.

### 3.2 Molecular modeling of *P. falciparum* 26S proteasome subunit Rpn2

**Figure 2:**
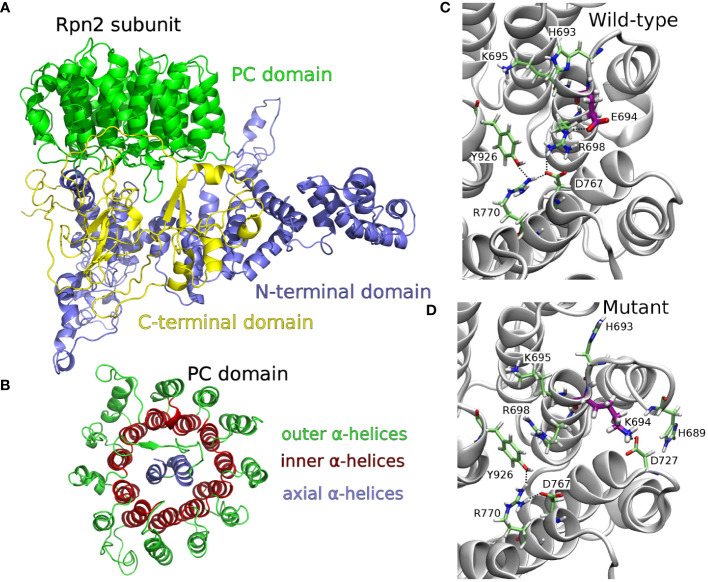
**(A)** 3D structure of the whole *P. falciparum* protein 26S proteasome, Rpn2 subunit, PC domain, obtained by threading method. **(B)** Top view focusing the toroidal-barrel-like PC repeat domain structural organization, highlighting outer, inner, and axial α helices. **(C)** Snapshoot of the MD simulation of wild type Rpn2 subunit (top view of the toroidal-barrel-like PC domain), highlighting E694 network interactions: salt bridge among R698 (inner helix) - E694 (inner helix) - D767 (inner helix) and salt bridge among R770 (inner helix) - D767 (inner helix) - Y926 (axial helix). **(D)** Snapshoot of the MD simulation of E694K mutant Rpn2 subunit (top view of the toroidal-barrel-like PC domain), highlighting the conformational change of R698 and the K694 network interactions: cation-π interaction between R698 and Y926; hydrogen bond between K694 and D727 or K694 and H689; salt bridge between D767 - R770 - Y926.

The 26S proteasome comprising a 20S proteolytic core and two 19S regulatory particles (19S-RP) plays a pivotal role in the proteolysis of ubiquitylated proteins in the cell. The 19S-RP subunits Rpn1 and Rpn2 are responsible for recognizing ubiquitylated proteins and priming them for proteolysis by the 20S core. The Rpn2 subunit is assembled from a rod-like N-terminal domain, a toroidal proteasome/cyclosome (PC) domain, and a globular C-terminal domain (He et al., 2012) (**Figure 2A**). The PC repeat domain is a concentric arrangement of α helices: two antiparallel α helices (axial helices), wrapped in a double layer of α helices, consisting of the inner and outer helices (**Figure 2B**). We built the wild-type structure of the *P. falciparum* Rpn2 subunit based on *P. falciparum* primary sequence (Uniprot ID Q8IKH3), through a fold recognition (threading) approach. Analysis of the model’s Ramachandran plot (**Supplementary Figure 1**) revealed that 87.2% of the residues occupy the most favorable regions which is close to the ideal of 90% (Laskowski et al., 1993), suggesting that the built model is acceptable. Based on wild-type structure modeled, the E694K mutant was computationally built. This mutation on *P. falciparum* corresponds to the E738K mutation on the PCHAS_133430 gene of *P. chabaudi* (**Supplementary Figure 2**). Considering the Rpn2 wild-type tertiary structure, we observe that E694 is surrounded by positively charged residues, such as H689, H693, K695 and R698. Hence, the substitution of glutamate with lysine (E694K) leads to a significant alteration in the residue’s standard interaction network.

To gain a deeper understanding of the dynamic behavior of the Rpn2 subunit in both the wild-type (WT) and E694K mutant (MT) models, we conducted two independent molecular dynamics simulations (MDs) (100 ns each, in duplicate) of the WT and MT structures. **Figures 2C, D** present snapshots of the most prevalent E694 network interactions (WT-MD) and K694 network interactions (MT-MD), respectively, as observed during the MD simulations. Hydrogen bond analysis of residues interaction, in WT and MT-MD simulations, are available as **Supplementary Data** (**Supplementary Figure 3**). In WT-MD simulations, E694 network interactions were extended from inner to axial helices, through salt bridges (**Figure 2C**). In MT-MD simulations, K694 interacts with outer helices residues/water molecules, and there is a rearrangement of neighborhood residues (conformations and interactions) (**Figure 2D**). The residue R698 underwent a conformational change, deviating from its WT state by disrupting interactions with inner helix residues and establishing new associations with axial helix residues (**Figure 2D**). This shift in conformation affects the interplay between the axial-inner and inner-outer α helices within the proteasome/cyclosome (PC) domain, subsequently influencing the toroidal organization of the PC domain.

## 4 Discussion

We characterized the genome-wide variations occurring after experimental evolution of resistance to artesunate + mefloquine in the rodent malaria parasite AS-ATNMF1, which was derived from the low artesunate-resistant parasite AS-ATN. This identified novel mutations in AS-ATNMF1, some of which were shown to be already present in is progenitor AS-ATN. Previously, we have shown that *P. chabaudi* AS-ATNMF1, selected for resistance to artesunate + mefloquine, has gained an extra copy of the multi-drug resistance 1 (*mdr1*) gene (Rodrigues et al., 2010) and, interestingly, that this does not incur in a fitness cost for AS-ATNMF1 (Rodrigues et al., 2013). Overall, this provided early evidence that *mdr1* amplification plays a role in resistance to the combination of these drugs in *P. chabaudi* (Rodrigues et al., 2010). These results have a parallel in *P. falciparum*, where *mdr1* amplification is associated with increased *in vitro* resistance or delayed parasite clearance after treatment with ATN and MF either alone or in combination (Price et al., 1997; Pickard et al., 2003; Price et al., 2004; Wongsrichanalai and Meshnick, 2008; Chaijaroenkul et al., 2010; Chavchich et al., 2010; Kubota et al., 2022).

It has been demonstrated in cancer cells that P-gp1 (coded by *mdr1*) is targeted to degradation by the proteasome and that increased ubiquitination results in reduction of P-gp1 function (Zhang et al., 2004). As shown in a previous study, the AS-ATN clone harbours a V2697F mutation in *pcubp1* gene, which is believed to affect the function of this protein, reducing de-ubiquitination of different proteins, which would probably increase protein degradation, including parasitic P-gp1, via the 26S proteasome (Hunt et al., 2007). Since AS-ATNMF1 has inherited this mutation it could be expected that reduced function of *pcubp1* product would cause an increase in P-gp1 degradation, therefore, being responsible for a reduction of P-gp1 expression. Interestingly, we found here that a previously undescribed E738K mutation in the 26S proteasome regulatory subunit Rpn2 was selected for with artesunate (in AS-ATN) and retained in AS-ATNMF1.

Our molecular modeling simulations suggest that the E694K mutation may disrupt axial-inner helix interactions and lead to the formation of new inner-outer helix interactions within the structure of the 26S proteasome regulatory subunit Rpn2. Consequently, this mutation has the potential to alter the organization of the toroidal PC domain and impact the recognition of ubiquitinated proteins. It is well-established that oxidative stress, as induced by artemisinin, can induce misfolding in proteins, which are subsequently degraded by the proteasome. We therefore speculate that the mutation in the 26S proteasome regulatory subunit Rpn2 may contribute to altering oxidation-dependent ubiquitination of P-gp1 and/or other artesunate protein targets, resulting in changes in protein turnover. These results re-enforce the overall notion that differential protein ubiquitylation is central to the biology of resistance to artemisinins in malaria parasites.

Interestingly, genetic mutations in a gene encoding a putative Kelch protein, K13, which is not mutated in *P. chabaudi*, are the most important resistance artemisinin determinants in natural populations of *P. falciparum* (Ashley et al., 2014). There is strong evidence for an endocytic pathway concentrated around a K13‐compartment, where apart from K13, a number of other proteins are located, including UBP1 and AP2-mu (Behrens et al., 2021). Also, it has been recently shown that reduced activity of Kelch13 and its interactors in this compartment causes a reduction in hemoglobin endocytosis and, consequently, the activation of artemisinins, resulting in parasite resistance (Birnbaum et al., 2020). In light of these observations, we hypothesize that 26S proteasome regulatory subunit Rpn2 may contribute to altered K13 turnover. In AS-ATNMF1 apart from the previously identified duplication and overexpression of the *pcmdr1* gene (Rodrigues et al., 2010), two additional SNPs were identified: (a) the A805,659T substitution in PCHAS_132020 in chr 13; and (b) the G1,155,448T in the PCHAS_143160 gene in chr 14 (**Table 1**). Interestingly, PCHAS_132020 and the orthologous *P. falciparum* protein PF3D7_1453200 possess an RNI domain. Fbox proteins with RNI domains are part of Skp1-Cullin-Fbox (SCF) complexes that ubiquitinate proteins with different substrate specificities (Jin et al., 2004). The PCHAS_132020 mutated residue 998 is located in the RNI domain (805-1035), which may lead to loss of Fbox-like function in this protein. The NEDD8 Cullin-activating protein inhibitor MLN4924 antagonizes the antimalarial activity of dihydroartemisinin (Bridgford et al., 2018); hence, chemical inhibition of SCF may phenocopy genetic loss of function in Fbox and other parasite SCF proteins. SCF mutations that reduce protein ubiquitination during normal parasite development may reduce the load on the proteasome when the parasite is exposed to artemisinin. In the future, inferring potential roles for the mutations reported here in the resistance phenotype will benefit from conformational studies, such as gene editing and drug sensitivity laboratorial assays comparing with wild-type parasites from the same genetic background, and phenotype-genotype association studies in natural parasite populations of human malaria.

## 5 Conclusions

We had previously shown that the evolution of resistance to the combination of artesunate + mefloquine selects malaria parasites with amplification of the *mdr1* gene. Additionally, we have now shown that ACTs select point mutations in other genes, including the 26S proteasome subunit. Molecular modeling calculations suggest that compared to the wild-type, the E694K mutant of the 26S proteasome Rpn2 subunit may alter the organization of the proteasome/cyclosome domain and this could impact in the recognition of ubiquitinated proteins. The present work lends weight to the general evidence that resistance to artemisinins is largely conferred by ubiquitin-proteasome-dependent protein turnover and provides novel molecular markers that may be tested in natural populations of human malaria parasites.

## Data availability statement

The datasets presented in this study can be found in online repositories. The names of the repository/repositories and accession number(s) can be found below: https://www.ncbi.nlm.nih.gov/genbank/, OR913732; https://www.ncbi.nlm.nih.gov/, SAMN39483236; https://plasmodb.org/plasmo/app/record/dataset/DS_5dab6e4a9f, GCA_900002335.2; https://www.ncbi.nlm.nih.gov/, OR913732.1.

## Ethics statement

The animal study was approved by Ethics Committee of the Instituto de Higiene e Medicina Tropical of Lisbon, Portugal, under PARECER 2/2006 from August 1st, 2006. The study was conducted in accordance with the local legislation and institutional requirements.

## Author contributions

GC: Writing – original draft, Writing – review & editing. AM: Data curation, Formal analysis, Methodology, Writing – review & editing. MM: Formal analysis, Methodology, Writing – review & editing. BN: Formal analysis, Methodology, Writing – review & editing. CA: Formal analysis, Funding acquisition, Supervision, Writing – review & editing. PF: Conceptualization, Formal analysis, Writing – review & editing. PC: Conceptualization, Formal analysis, Funding acquisition, Investigation, Supervision, Writing – original draft, Writing – review & editing.
