# Supplementary material for: Whole genome sequencing identifies novel mutations in malaria parasites resistant to artesunate (ATN) and to ATN + mefloquine combination
Source: Front Cell Infect Microbiol. 2024 Mar 1;14:1353057. doi: 10.3389/fcimb.2024.1353057 (PMC10940360; doi:10.3389/fcimb.2024.1353057)
Supplement: Supplementary file 1 [file DataSheet_1.pdf]

Supplementary Table 1. List of oligonucleotide primers used for PCR amplification and di-deoxy sequencing verification of a sub-set of mutations identified by SOLEXA in AS-ATNMF1.

GENE (position)		SEQUENCE	ANNEALING TEMPERATURE
3'-PCAS_011300	sense	GCA TCG ATC ATG TGA TAA ACC	52ºC
	anti-sense	GAT GTT TGC TGA CTC GCA CA	
PCAS_020900	sense	GGT ATG ATC CAT TAT CTT GTT CG	51ºC
	anti-sense	TGT ATC AGG ATC TAG TAG TTC G	
PCAS_030400	sense	GAA CCT TCC CGA TAA TTT TGA	48ºC
	anti-sense	TAT GTA TTT ATC TTC CCT GAG TTG	
PCAS_030230	sense	CTA AAC CTG AAC CTA AAC CAT	52ºC
	anti-sense	CAG GAC GAA GGT GCA TCA TGG C	
PCAS_031370	sense	GCT TGC TTA TGC ATA CTT CG	53ºC
	anti-sense	TCG CGA AAT CGA ACT TAA AAT AC	
PCAS_031520	sense	GAG GGT TTA TAG TGT TTG TAT C	54ºC
pos. 523111 to 523127	anti-sense	CAT TCA AAC TCA TCA GCC TTA G	
PCAS_031520	sense	GAA CAG ACA TTT CTT GAT TTT GC	54ºC
pos. 524946 to 524964	anti-sense	CTA TTC AGG CAA TGG TAA CC	
3'-PCAS_061710	sense	CAT TCA GCT TGT AAC TTG GG	56ºC
	anti-sense	GAG AAG GTA TTA TGG TTC CTG	
PCAS_070250	sense	CCC GCC CCA GCA AAA TAA AC	54ºC
	anti-sense	GCT GAA AAG AAA GCG GAA GAG	
PCAS_072830	sense	TAA ATC TTC ATT GAT ATC TGG	51ºC
	anti-sense	CAT TCC CAA GTC CCT TAA AA	
34 bp deletion chr 7	sense	CGT ATA AAG GCT GTG ACA AC	52ºC
	anti-sense	CAG GTT CGT TTT GTA CAT ACC	
PCAS_083110	sense	GAC AAA ACA AAA AAG AAA TAA CAC	48ºC
	anti-sense	ACA TGG TTA TTA TGG GTT TTA GTA	
5'-PCAS_083770	sense	GAA AGT GAA GCA CCA TTC GG	56ºC
	anti-sense	CAC TGT GTC ATA TTC AAG ATT TG	
5'-PCAS_092710	sense	GCA TGC ATG TAG ATC ATA ACA C	54ºC
	anti-sense	GTT TAC ATT CTT CGC TGC TAT C	
PCAS_093250	sense	CGT GTT GGA AAA TGA TGA AA	49ºC
	anti-sense	CGA AGG AAA AGG AGT GAG TAA G	
PCAS_110620-5	sense	CGG TAT GTG TGT ATA TGT AC	48ºC
	anti-sense	CAT TTT GTT GGC TTA TTT ATG TC	
PCAS_111100	sense	GAA CCG TAT CAT TAA TTC CTG G	52ºC
	anti-sense	GGT GGA AAT AAT TCA ACA ACT ATG	
PCAS_121630	sense	CCC TTT ATC ACC CGA ATA TTT C	55ºC
	anti-sense	CAT ATT TTT TAA GGG GTA AGT GC	
PCAS_130140	sense	CTC TGG GTC CGA AAT CAA TG	51ºC
	anti-sense	GTC ACC ATT AGG CCC ATA AG	
PCAS_133430	sense	GGT TGT TGT TTA GGA TTA GGT C	49ºC
	anti-sense	CAT ACC ACC ATA TCT GAT TAT TG	
PCAS_132020	sense	CCA GAT ATT AAG CAA TAC AGC AC	56ºC
	anti-sense	CGG CCA CAC ATA TAT TAA GGA	
PCAS_143160	sense	GAG GAC GCG AAA AGG AAA G	53ºC
	anti-sense	TCT CCT CCA CAC TAT CAG C	
PCAS_144430	sense	TCA TTG CTG TTG GTA ACT TCA	53ºC
	anti-sense	AGA CTG ATG GTA AGG GCG A	

Supplementary Table 2. SNPs identified by whole-genome re-sequencing of AS-ATNMF1. Mutations with high confidence of representing true SNPs are highlighted in green, intermediate level, highlighted in orange and low levels of confidence highlighted in red.

			AS-WTSI		AS-SENS					ATNMF-1							
Chromosome	Type	Analysis	End	Base	Base	Read depth	Main Base	Relative coverage	Relative coverage	Base	Read Depth	SSAHA/MAQ Quality	Gene ID	Aminoacid Substitution	Nearest gene	Gene abbreviation	Annotation
2	SNP	MAQ/SSAHA	217 047	C	C	56	C	1,4	0,3	A	13	62	PCHAS_020720	**V2697F**		UBP1	deubiquitinating enzyme
6	SNP	MAQ/SSAHA	636 862	A	A	19	A	0,5	0,3	G	14	70	x	x	3-PCHAS_061710		seryl-tRNA synthetase
7	SNP	MAQ/SSAHA	994 546	G	G	67	G	1,7	0,7	A	34	99	PCHAS_072830	**S109N**		DHFR	dihydrofolate reductase
11	SNP	MAQ/SSAHA	996 332	G	G	56	G	1,4	1,6	T	80	99	PCHAS_112780	**A173E**		AAT1	aminoacid transporter
13	SNP	MAQ/SSAHA	805 659	A	A	32	A	0,8	0,1	T	3	15	PCHAS_132020	**K998L**			conserved Plasmodium protein
13	SNP	MAQ/SSAHA	1 322 938	G	G	35	G	0,9	1,7	A	85	99	PCHAS_133430	**E738K**			26S proteasome subunit
14	SNP	SSAHA	936 945	T	T	13	T	0,3	0,1	G	4	20	x	x	5-PCHAS_142600		conserved Plasmodium protein
14	SNP	MAQ/SSAHA	1 155 448	C	C	46	C	1,2	1,7	A	84	99	PCHAS_143160	**D560Y**			conserved Plasmodium protein
7	SNP	SSAHA	689 803	A					0,1	T	3	15					
13	SNP	SSAHA	1 792 583	C						T	10	2					
bin	SNP	SSAHA	247 620	T					0,1	C	3	6	x		5.PCHAS_000660		Pc-fam
bin	SNP	SSAHA	411 999	T					0,1	C	3	8	x		PCHAS_001050-3		CIR
1	SNP	SSAHA	483 843	T					0,1	G	5	12	x		3-PCHAS_011300		conserved Plasmodium protein
3	SNP	MAQ/SSAHA	82 406	T	T	11	T	0,3	0,2	G	10	20	x		PCHAS_030230		Plasmodium exported protein
7	SNP	MAQ/SAAHA	876 917	A	A	54	A	1,4	0,3	G	16	80	x		PCHAS_072420-3		conserved malaria protein
7	SNP	MAQ/SSAHA	876 919	C	C	54	C	1,4	0,3	A	17	85	x		PCHAS_072420-3		conserved malaria protein
11	SNP	SSAHA	214 749	A					0,2	C/A	11	17	x		PCHAS_110620-5		rhomboid protease
11	SNP	SSAHA	415 965	A					0,1	C	5	12	PCHAS_111100				myo-inositol 1-phosphate synthase
12	SNP	SSAHA	566 547	A					0,3	G/A	14	27	PCHAS_121630				40S ribosomal protein S3A
13	SNP	MAQ	56 840	G	G/A	52	G	1,3	0,1	A	6	45	PCHAS_130140				HAD hydrolase, putative

Supplementary Table 3. Indels and CNVs identified by whole-genome re-sequencing of AS-ATNMF1. Mutations with high confidence of representing true indels/CNVs are highlighted in green, intermediate level, highlighted in yellow and low levels of confidence highlighted in red. The indel highlighted in blue represents a false negative and was identified by di-deoxy sequencing.

	AS-WTSI						AS-50SP				ATNMF-1				
	Chromosome	Type	Analysis	Start	End	Size	Read depth	Relative coverage	Comparative coverage	Relative coverage	Read Depth	SSAHA/MAQ Quality	Gene ID	Nearest gene	Annotation
1	**3**	Deletion	SSAHA	472 273	472 275	3						36/43	PCHAS_031370		conserved Plasmodium protein
2	**12**	CNV	SSAHA	1 382 067	1 462 751	80 685	43	1,0	2,0	1,9	96		PCHAS_123820		80 Kb amplification
3	**13**	Deletion	SSAHA	56	60 867	60 812	25	0,6	0,0	0,0	0		PCHAS_130140		60 Kb deletion
4	**7**	Deletion	SSAHA	876 894	876 927	34							x	PCHAS_072420-3'	conserved malaria protein (34 bp deletion)
5	**5**	indel	SSAHA	682 682	685 106	2 425	186	4,2	0,2	0,9	44		PCHAS_051920		S-Antigen
6	**bin**	Deletion	SSAHA	305 928	339 914	33 987	31	0,7	0,0	0,0	0		PCHAS_000890		80 Kb deletion including mdr1 gene
7	**2**	indel	SSAHA	301 741	301 767	27	20	0,5	0,2	0,1	5		PCHAS_020900		ubiquitin carboxyl-terminal hydrolase
8	**3**	indel	SSAHA	137 023	137 033	11	53	1,2	0,2	0,3	15		PCHAS_030400		conserved Plasmodium protein
9	**3**	indel	SSAHA	523 111	523 127	17	21	0,5	0,1	0,1	3		PCHAS_031520		vacuolar protein-sorting protein VPS45
10	**3**	indel	SSAHA	524 946	524 964	19	26	0,6	0,2	0,1	5		PCHAS_031520		vacuolar protein-sorting protein VPS45
11	**7**	indel	SSAHA	103 522	103 536	15							PCHAS_070250		erythrocyte binding protein 1
12	**8**	indel	SSAHA	1 140 891	1 140 910	20	42	0,9	0,2	0,2	10		PCHAS_083110		leucine-rich repeat protein 8
13	**8**	indel	SSAHA	1 355 069	1 355 081	13							x	5'-PCHAS_083770	haloacid dehalogenase-like hydrolase
14	**9**	Deletion	SSAHA	919 771	919 771	1						2/3	x	5'-PCHAS_092710	26S proteasome regulatory complex subunit
15	**9**	Deletion	SSAHA	1 113 660	1 113 660	1						14/16	PCHAS_093250		conserved Plasmodium protein
16	**14**	indel	SSAHA	1 595 169	1 595 276	108							x	3'-PCHAS_144430	Cyclin-related protein

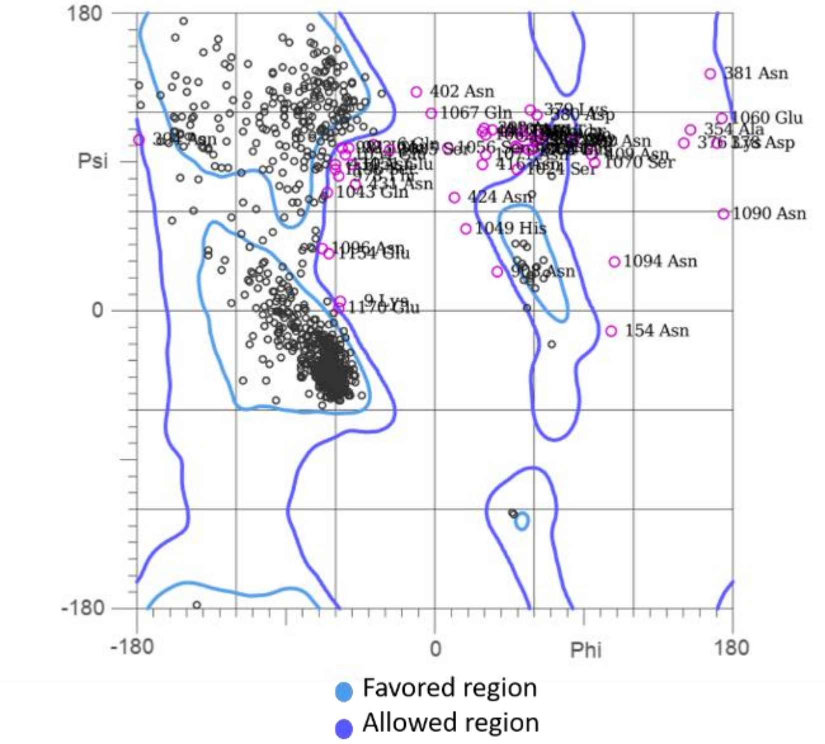

Supplementary Figure 1. Ramachandran plot of the 3D model of the wild-type *P. falciparum* 26S proteasome subunit Rpn2. No residues of the toroidal PC domain are in forbidden regions on the Ramachandran plot.

```
P.chabaudi  MDVEKTIHENDIVTSASGVIALLNEEDASLKIFGLEKLNSVVDIYWPELADYIFKIEELCEDEEFSGRELANLVASKVYYHLEKYPEALKYALCAGKLFNINEKSQYIETMLAKCIEKYVEIREKDYEGV
P.f 3D7     MRFENQFSKHDIVTSASGVIALLNEEETSLKLFGLEKLNAIVDVYWPELADSIFKIEELCEDEDFVGRELANLLASKIYFHLEKYSEALKYALCAGKLFNIKEKSQYVETMLAKCIEKYVEIREMQYD--

            131                                                                                                                    260
P.chabaudi  DPNSTGRNNAINYSTNENGFSSSYTLNTNYNNIDPEYKKGKNELNTQNNIEDHNRNIYDSYDYNGKGINANSSTKNNNILFSRDNENDKVKEENNIFKDDLNNDINKKMEIFVDDMLEICIKNNSIKEAL
P.f 3D7     ----------IDYNNQHN-------INDGITN---------------------NNDIFNAY--------------NNNDLVELNSHNELLNKETDIFRFDINNEINQKMELLVDEMIDVCIKSNDIKEAL

            261                                                                                                                    390
P.chabaudi  GVALDARRLDKVEYIILNAPNKLEILQHSISNERHINTTKKFRNDFFKLLVKIYLSMSEEEIKYEYVNLCECLFYINDYKTVAEILLKLIENYHLMVYQISFDLVDLENINFLKNILKEIKEIIIKNKSY
P.f 3D7     GVALDARRLDKVEYIIANAENKIEILAHSINNEKHINMNKSFRNEYFKLLVNLYLSLSEEELKTEYINLCECLYYIDDYKKVAEILLKLLHNYHLMAYQISFDLVDFENKIFLRNILGQIKENLIQNKAY

            391                                                                                                                    520
P.chabaudi  YYGEENY-----KHLNPDLLKKNSSNSAISEGTNEGV-KSENTSEQDAQKENSQDSSNTENAQDNSEGTDSANPTSTAVN----TDDKKNAKDNEEKDKIPEDVLMYVNEQHHLYEKIKKLIFILTGKIT
P.f 3D7     YFGEEYFVTTPQEENNDADNKENESNAITNEQQDENNNEKEDKDNNDGKNPEHNDDNNKPNDNLTNDEPTYNNNDDKAINDGNDNDNNNNNINNNNNNIVEDVLKYISKKHIFYNKMKKLLYILTGKVT
            521                                                                                                                    650

P.chabaudi  TSLYIEFLHRNNHADLILLDSYKNVIDSRSSITHHGIVIAHGLMQAGTTCDVFLRSNIEWLSKAVNWAKFSSTASLGVVYKGHVNESFMVLSSHLPYNDISRQITNNINANISQSDVYSESGSLYALGLI
P.f 3D7     GNLYIEFLHRNNHADLILLDTYKNIVDSRSSITHHGIVIAHALMQTGTTCDVFLRSNIEWLSKAINWEKFSATASLGVVYKGHVNESFMVLSTHLPYNDVSTEIANNINVGLAPSGVYSEGGSLYALGLI

            651                                                                                                 738                 780
P.chabaudi  HATYNTNYKKVRDFLLSQLKGSNNNEVLQHGCCLGLGLVCLEQNDDEQVYDELKSVMYSDSAVAGESAAYGIGLLKLGSGDEKCVDELLAYAHDTQHEKITRACSISLGFVMFQKEREADNLIEELINDK

P.f 3D7     HANYNTNDKKVKNYLMSQLKSNMNDEVLQHGCCLGLGLVCLGDSNDENTYDELKAILYSDSAVAGESAAYAIGLLKLGSGDDKCIDELLAYAHDTQHEKITRACSISLGFVMFQKEREADSLIEELVSDK
                                                                                                                694

            781                                                                                                                    910
P.chabaudi  DAIIRYGGMFTIALAYCGLSNYNKHVIKKLLHFSVSDVSDDVRRAAVIALGFVLCNTPAQVPMFLNLLIESYNPHVRYGAALALGIACAATGNEEAVNMLMPLLTDTTDFVRQSAFISLGLIFQQSNENV
P.f 3D7     DAIIRYGGMFAIAMAYCGLSNCNKHIIKKLLHFSVSDVSDDVRRAAVIALGFVLCNSPNQVPKFLNLLIESYNPHVRYGAALALGIACSATANEEAINMLMPLLTDTTDFVRQSAFISLGLIFQQSNEHC

            911                                                                                                                   1040
P.chabaudi  NPNFKKFKDEIMKILSDKHEDIIAKFGATVGLGLLDICGRNAISTFFTRRANIIRPQSAVGFCLFCQLWYWFPLIHMISLTFLPTCLIGLTEDLKVPKNFTILSTKN-QAFDYPSFLSKEKVQEKKETVT
P.f 3D7     NPNFKKYKEEIMKILSDKHEDIIAKFGAIVSAGLLDICGRNAISTFFTRRGNIIRPQAAAGFCLFSQLWYWFPLIHMISLTFMPTCLIGLTEDLKVPKNFSILSTSNNQNFDYPSFLSKEKTQEKKETVT
            1041                                                                                                                  1170

P.chabaudi  AILSTTDKRKSLKLKKQKNENKLTKEKNPQDDSSSVLSDGKSMKNLEILSTAATIGQSSHVSHAESVEGSANDENSNDHQNDANQFSQLQRIKKSD-KSKSASL--SHATTVDMKNPCRVIKTQEKYIEY
P.f 3D7     AVLSTTAKRKTLKLKKQKNDSKITKERTAQDDNSSVLSDGKSMKNLEILSTAATIGQSSHVSHAESVEGSANDENSHDHAQDGSNISNVQKGKKSEAKSKSGANENSNNNTVDMKNPCRVIKMQEKYIEY

            1171                                                    1226
P.chabaudi  PPNSRFKPIISIRKSGFIMLSDTTPTEPFDFIEPKLESGNKKEVPPFEPFTWKDEN
P.f 3D7     IPNSRYIPVLPTRKSGFIMLNDITPLEPSECIEINFEETSKKEAPPFEPFSWKEEN
```

Supplemental Figure 2. Pairwise alignment between the wild-type sequences of the 26S proteasome regulatory subunit RPN2 proteins of *Plasmodium chabaudi* (PCHAS_1334300) and *Plasmodium falciparum* (PF3D7_1466300), highlighting the conserved glutamic acid (**E**) at residues 738 and 694, respectively. Made using the tool "Multiple sequence alignment with hierarchical clustering". F. CORPET, 1988, Nucl. Acids Res., 16 (22), 10881-10890" (http://multalin.toulouse.inra.fr/multalin/).

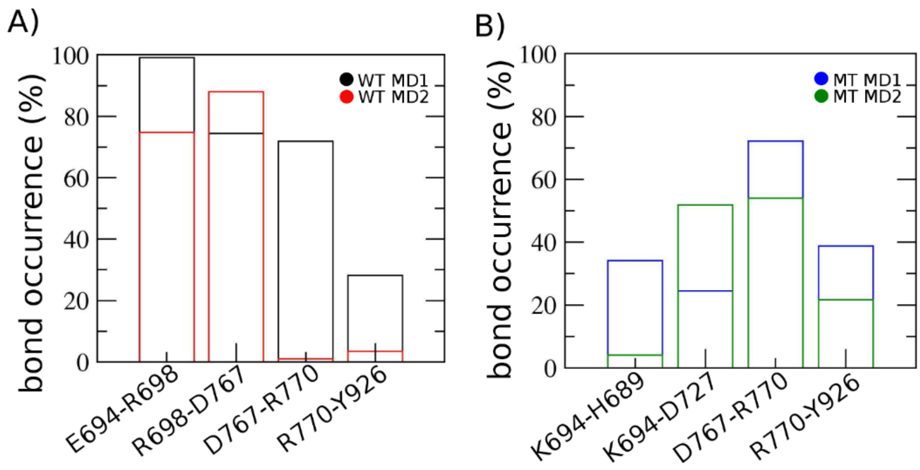

Supplementary Figure 3. Hydrogen bond frequency occurrence during MD simulations of the **(A)** wild-type and **(B)** E694K mutant of 26S proteasome subunit Rpn2.
